# Structure–Function Analysis of the Non-Muscle Myosin Light Chain Kinase (nmMLCK) Isoform by NMR Spectroscopy and Molecular Modeling: Influence of *MYLK* Variants

**DOI:** 10.1371/journal.pone.0130515

**Published:** 2015-06-25

**Authors:** Kui Shen, Benjamin Ramirez, Brandon Mapes, Grace R. Shen, Vijay Gokhale, Mary E. Brown, Bernard Santarsiero, Yoshitaka Ishii, Steven M. Dudek, Ting Wang, Joe G. N. Garcia

**Affiliations:** 1 Institute for Personalized Respiratory Medicine, Section of Pulmonary, Critical Care, Sleep, and Allergy, University of Illinois at Chicago, Chicago, Illinois, United States of America; 2 Center for Structural Biology, University of Illinois at Chicago, Chicago, Illinois, United States of America; 3 College of Pharmacy and BIO5 Institute, University of Arizona, Tucson, Arizona, United States of America; 4 Arizona Respiratory Center and Department of Medicine, University of Arizona, Tucson, Arizona, United States of America; University of Chicago, Department of Medicine, UNITED STATES

## Abstract

The *MYLK* gene encodes the multifunctional enzyme, myosin light chain kinase (MLCK), involved in isoform-specific non-muscle and smooth muscle contraction and regulation of vascular permeability during inflammation. Three *MYLK* SNPs (P21H, S147P, V261A) alter the N-terminal amino acid sequence of the non-muscle isoform of MLCK (nmMLCK) and are highly associated with susceptibility to acute lung injury (ALI) and asthma, especially in individuals of African descent. To understand the functional effects of SNP associations, we examined the N-terminal segments of nmMLCK by ^1^H-^15^N heteronuclear single quantum correlation (HSQC) spectroscopy, a 2-D NMR technique, and by *in silico* molecular modeling. Both NMR analysis and molecular modeling indicated SNP localization to loops that connect the immunoglobulin-like domains of nmMLCK, consistent with minimal structural changes evoked by these SNPs. Molecular modeling analysis identified protein-protein interaction motifs adversely affected by these *MYLK* SNPs including binding by the scaffold protein 14-3-3, results confirmed by immunoprecipitation and western blot studies. These structure-function studies suggest novel mechanisms for nmMLCK regulation, which may confirm *MYLK* as a candidate gene in inflammatory lung disease and advance knowledge of the genetic underpinning of lung-related health disparities.

## Introduction

The responses of pulmonary endothelial cells (ECs) to external stimuli, including bioactive agonists and mechanical stress, largely proceed through the spatially-targeted rearrangement of the dynamic, functionally complex actin cytoskeleton [1, 2]. As a critical actin-binding protein and driver of actin cytoskeletal rearrangement [3], in lung endothelium, the calcium/calmodulin-dependent non-muscle isoform of myosin light chain kinase (nmMLCK) is required for vascular barrier regulation, fluid balance, trafficking of inflammatory cells, and vascular responses to mechanical stretch [1–7].

During acute lung injury, spatially-localized actomyosin contraction and increased stress fiber and paracellular gap formation results in profound loss of barrier integrity and increased vascular permeability, a defining feature of inflammation. Phosphorylation of myosin light chains (MLCs) on Ser^19^ and Thr^18^ catalyzed by nmMLCK enables ratcheting of actin and myosin bonds in these processes [8–11]. Strategies that reduce nmMLCK activity decrease edema-promoting agonist–induced cell contraction and barrier disruption *in vivo* and *in vitro* [11, 12] as well as attenuate leukocyte diapedesis [4, 5]. Conversely, nmMLCK is also essential to lung vascular barrier protection and recovery induced by barrier-enhancing or barrier-restoring agonists such as hepatocyte growth factor [13] and sphingosine 1-phosphate (S1P) [14]. These agonists dramatically drive rapid and spatially-distinct increases in actin polymerization and MLC phosphorylation that are confined to lamellipodial membrane protrusions and the cortical actin ring, resulting in closure of inflammation-mediated paracellular gaps thereby restoring barrier integrity [13–15]. This dual role for nmMLCK in critical lung processes highlights the need to understand spatiotemporal regulatory mechanisms for this key multi-functional enzyme.

We, and others, have demonstrated that reversible Ser/Thr and Tyr nmMLCK phosphorylation contributes to differential regulation of enzymatic activity and cellular spatial targeting of the kinase [2, 8, 16]. For example, cAMP-dependent protein kinase A (PKA)–mediated phosphorylation of nmMLCK exerts paradoxical effects on kinase activity depending on whether the Ser/Thr phosphorylation sites resides within the unique nmMLCK N-terminus, the putative Src homology (SH) 3-binding sequences, or the calcium/calmodulin binding region [4, 17, 18]. We have also previously identified Tyr phosphorylation of nmMLCK by pp60src or c-Abl during barrier disruption [8, 19], during recovery as well as following exposure to barrier-enhancing stimuli [2, 16]. These studies indicated that pp60src- or c-Abl-mediated phosphorylation of nmMLCK at Y^464^ and Y^471^ increases MLC kinase activity [2, 8]. Similarly, inhibition of Tyr phosphatase activities results in significant accumulation of pTyr-containing nmMLCK, increased MLC kinase activity, endothelial cell contraction and barrier dysfunction [20, 21]. In addition to nmMLCK regulation by reversible Tyr phosphorylation, these studies suggest that the N-terminal SH2 and SH3 domain-binding motifs (including an SH2-binding motif containing phosphorylated Y^464^) regulate nmMLCK interactions with other cytoskeletal regulatory proteins.

**Fig 1:**
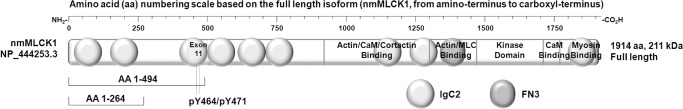
Selection of the N-terminal segments of nmMLCK1. The segment of 1-494aa was initially selected for protein expression within the N-terminal sequence of nmMLCK1, containing three ALI-associated SNPs and two phosphorylatable Tyr sites, Y464 and Y471. This sequence generates a protein of ca. 53 kDa, within a suitable range of size for practical bacterial protein expression and survived preliminary NMR trials. Included in this ca. 500aa protein are three immunoglobulin C-2 type (IGc2) domains and a low-complexity region (preceding the 3rd IGc2 domain) as predicted by SMART. Subsequently, a shorter 1-264aa segment of ca. 28 kDa was also generated spanning the three ALI-associated SNPs (two IGc2 domains) and exhibited advantages for NMR-based structural determination.

The *MYLK* gene encodes several MLCK isoforms including nmMLCK and several nmMLCK variants that result from alternative splicing [22]. The nmMLCK isoform is distinguished by the presence of an extended N-terminus [23] containing structural elements that potentially participate in the spatiotemporal regulation of nmMLCK activities. Consistent with the role of nmMLCK in lung inflammatory processes, several amino acid-altering *MYLK* SNPs in the unique N-terminus of nmMLCK (P21H, S147P, V261A) are highly associated with the susceptibility to acute inflammatory states such as acute lung injury/acute respiratory distress syndrome (ALI/ARDS) and severe asthma, especially in individuals of African descent [24–26]. The two major nmMLCK splice variants, nmMLCK1 (ca. 211 kDa) and nmMLCK2 (ca. 203 kDa), differ by 69 amino acids within exon 11 (partially deleted in nmMLCK2, see Fig 1) [27], a stretch that contains the highly phosphorylated Y^464^ and Y^471^, suggesting that nmMLCK splice variants are differentially regulated by Tyr phosphorylation.

Despite the important role and potentially complex regulation suggested by previous studies for the SNP-embracing N-terminus of nmMLCK, a clear understanding of the molecular mechanisms and structural basis for these disease-SNP associations and the spatiotemporal regulation of nmMLCK variants remains elusive. In this study, we attempted to examine N-terminal segments of SNP variants of nmMLCK from a novel angle, by ^1^H-^15^N heteronuclear single quantum correlation (HSQC) spectroscopy, a 2-D nuclear magnetic resonance (NMR) technique, and by *in silico* molecular modeling.

## Materials and Methods

### DNA constructs and protein expression

The appropriate DNA sequences encoding the 1–494 amino acid (aa) and 1-264aa segments of nmMLCK, with codon optimization (Genscript, Piscataway, NJ) and desired SNP variations, were integrated by routine recombinant DNA techniques [28] into pTXB1 plasmid at the NdeI and SapI restriction sites in frame with intein-chitin-binding domain (CBD) (New England Biolabs [NEB], Ipswich, MA) and verified by sequencing, respectively. The plasmids were then transformed into *Escherichia coli* BL21 (DE3) competent cells (Agilent Technologies, Santa Clara, CA). Recombinant protein expression was induced at a final IPTG concentration of 0.5 mM at 298 K for 3 h or at 289 K for 16–20 h after cultures at 310 K reached an absorbance of 0.6–0.8 at 600 nm. LB broth (Fisher Scientific, Pittsburgh, PA) and ^15^N-enriched BioExpress cell growth media (Cambridge Isotope Laboratories, Andover, MA) were used in the bacterial cultures as appropriate depending on whether or not isotope labeling is needed.

### Protein purification and modification

Bacteria were harvested by centrifugation (6000 g) and the desired proteins were extracted as previously described with minor modifications [28, 29]. Briefly, the harvested *Escherichia coli* bacteria were lysed by passing through French press in the lysis buffer (25 mM Na-HEPES, pH 8.0, 150 mM NaCl, 1 mM MgSO_4_, 5% ethylene glycol, 5% glycerol) with 1 mM PMSF. The clarified cell lysates (ca. 40 mL) were loaded onto prewashed chitin column (ca. 5 mL) and the column subsequently washed with the column buffer (25 mM Na-HEPES, pH 7.0, 250 mM NaCl, 1 mM Na-EDTA, 0.1% Triton X-100) first (10x column volume) and the cleavage buffer (25 mM Na-HEPES, pH 8.0, 250 mM NaCl, 1 mM Na-EDTA) later (10x column volume). The chitin column was then washed with the cleavage buffer containing 50 mM DTT (5 mL) at 4°C and incubated with the same DTT-containing buffer (10 mL) at room temperature overnight (20 h). The eluates (cleavage buffer, ca. 10 mL each time) were then combined (ca. 30 mL in total) and concentrated to 1–3 mL, which was subsequently dialyzed against appropriate buffers for 2 days with at least ten buffer changes. The dialyzed protein was then concentrated to ca. 0.5–1 mL as necessary, which was used directly or stored in -80°C freezer. In the case of the C-terminal biotinylation, the chitin column was washed with the cleavage buffer containing 20 mM sodium 2-mercaptoethanesulfonate (Na-MESNA) instead of 50 mM DTT, and then incubated with the cleavage buffer containing 20 mM of Na-MESNA and 1mM of an N-terminal Cys-containing peptide that carried a biotinylated Lys (NEB #E6901). The dialysis buffer used for NMR sample preparation was 1x PBS, pH6.8, 1mM TCEP. The dialysis buffer for other purposes was the cleavage buffer containing 1mM TCEP. The purity of all the proteins was confirmed to be > 90% by SDS–PAGE and their molecular weights were confirmed by SDS–PAGE and MALDI-TOF MS. Protein yields were typically 5–10 mg per liter of culture.

For the C-terminal biotinylation and Tyr phosphorylation, two variants of the 1-494aa segments, wild type (P21-S147-V261)-1-494-biotin and a single SNP (P147)-1-494-biotin, were expressed and purified. The *in vitro* Tyr phosphorylation of the two segments by c-Abl was performed as previously described for nmMLCK [2]. Western blot was performed to confirm Tyr phosphorylation by the catalytic domain of c-Abl (Upstate Biotechnology, Lake Placid, NY). The wild type-1-494-biotin-pTyr was tested in a chip loading experiment in surface plasmon resonance (SPR) using the streptavidin (SA) sensor chip (GE Healthcare Biosciences, Piscataway, NJ).

For NMR experiments, we expressed and purified five variants of the 1-494aa segments, with single SNP or SNP combination, which included wild type-1-494-^15^N, P147-1-494-^15^N, H21-P147-1-494-^15^N, A261-1-494-^15^N, and H21-P147-A261-1-494-^15^N. We also generated two variants of the 1-264aa segments, wild type-1-264-^15^N, and P147-1-264-^15^N.

### Analysis of nmMLCK by NMR spectroscopy

The initial 1D proton NMR was performed with the unlabeled wild type-1-494. The ^1^H-^15^N HSQC experiments were performed with five variants of the AA 1–494 segments and two variants of the 1-264aa segments, respectively, on a Bruker 900 MHz NMR spectrometer (Center for Structural Biology, University of Illinois at Chicago). The experiments typically used 0.2–0.5 mM proteins in 1x PBS, pH 6.8, 1mM TCEP, and 10% D_2_O. Substantial care was exerted to ensure reliable comparison of all HSQC results on the SNP variants of varied lengths including using the same dialysis vessel if possible and strict use of well-defined buffers to achieve maximally similar conditions for all HSQC experiments.

All ^1^H-^15^N HSQC experiments were carried out at 25°C on a 900 MHz Bruker NMR spectrometer. The pulse sequence hsqcfpf3gpphwg was chosen. Pulse width was 14 μs for ^1^H and 45 μs for ^15^N, respectively. A total of 1024 complex points for an acquisition dimension and 256 complex points for an indirect dimension were recorded with 16 or 32 scans, acquisition time 20 ms per 2k data points, delay time 1s, and states-TPPI acquisition technique. The data were processed with 2k (f2) × 512 (f1), zero fill and no linear prediction. The resulting spectra have a sweep width of 14 ppm (centered on 4.7 ppm, H_2_O) for ^1^H and 36 ppm (centered on 118 ppm) for ^15^N.

### Cell signaling and imaging

Cell culture, S1P stimulation, and fluorescence imaging of the fixed and live cells were previously described with minor modifications [30]. The phosphorylation of Y^464^ in nmMLCK was detected by rabbit polyclonal antibody to pY^464^ in nmMLCK1 (Santa Cruz Biotechnology, Santa Cruz, CA) and goat anti-rabbit secondary antibody conjugated with Alexa Fluor 546 (Life Technologies, Grand Island, NY). Actin was detected by Alexa Fluor 633 Phalloidin (Invitrogen, Carlsbad, CA). Pull-down of nmMLCK was performed using anti-Flag M5 antibody (Sigma-Aldrich, MO) or anti-GFP antibody (Invitrogen, NY) as appropriate. 14-3-3 proteins were detected using pan-14-3-3 antibody (Cell Signaling Technology, MA).

### Molecular modeling

Protein homology modeling was performed using Swiss-Model [31]. Specifically, we identified the structures as optimal templates that span the region of interest within the N-terminus nmMLCK1 as well as possess the highest sequence identity, with the quality of model assessable by QMEAN Z-score [32]. The results were processed and visualized using PyMol (The PyMOL Molecular Graphics System, Version 1.5.0.4 Schrödinger, LLC.). The root-mean-square deviation (RMSD) values were obtained using SuperPose [33].

## Results

### Selection of the N-terminal segments of nmMLCK1

The segment of 1-494aa was initially selected for protein expression within the N-terminal sequence of nmMLCK1, containing three ALI- and asthma-associated SNPs and two Tyr phosphorylation sites, Y^464^ and Y^471^. This sequence generates a protein of ca. 53 kDa in average molecular weight, within a suitable range of size for practical bacterial protein expression and survived preliminary NMR trials. This protein contains three immunoglobulin C-2 type (IGc2) domains and a low-complexity region (preceding the 3rd IGc2 domain) as predicted by SMART [27, 34, 35] (Fig 1). Subsequently, a shorter 1-264aa segment of ca. 28 kDa was generated that also contains the three ALI-associated SNPs (two IGc2 domains) and exhibited advantages for NMR-based structural determination (Fig 1).

### Selection of the SNP variants of nmMLCK1

Our plan was to use a minimum number of proteins to study structure-function relationship of these nmMLCK1 SNP variants. Therefore we initially chose a limited number of such proteins (wild type-, P147-, H21-P147-, and H21-P147-A261-1-494aa, a half of all possible SNP combinations including wild type, single, double and triple SNP mutants) for NMR studies based on our previous studies that P147 SNP variant was more highly associated with ALI and asthma as well as on the assumption that 147P/S mutation may be directly involved in changing posttranslational modification (see below). Since H21-P147-1-494aa double SNP mutant was initially studied (giving preference over 21P/H SNP), an additional SNP mutant A261-1-494aa (giving preference over another, 261V/A, SNP) was also included to avoid bias and further verify our finding (see below) on the independent and local NMR signal changes associated with each SNP mutant. Similarly, we chose wild type-1-264aa and P147-1-264aa based on the potential involvement of 21P and 147S, the latter more directly, in posttranslational modification, as we analyzed below for 147S phosphorylation and 14-3-3 binding.

### Establishment of appropriate protein expression platform

**Fig 2:**
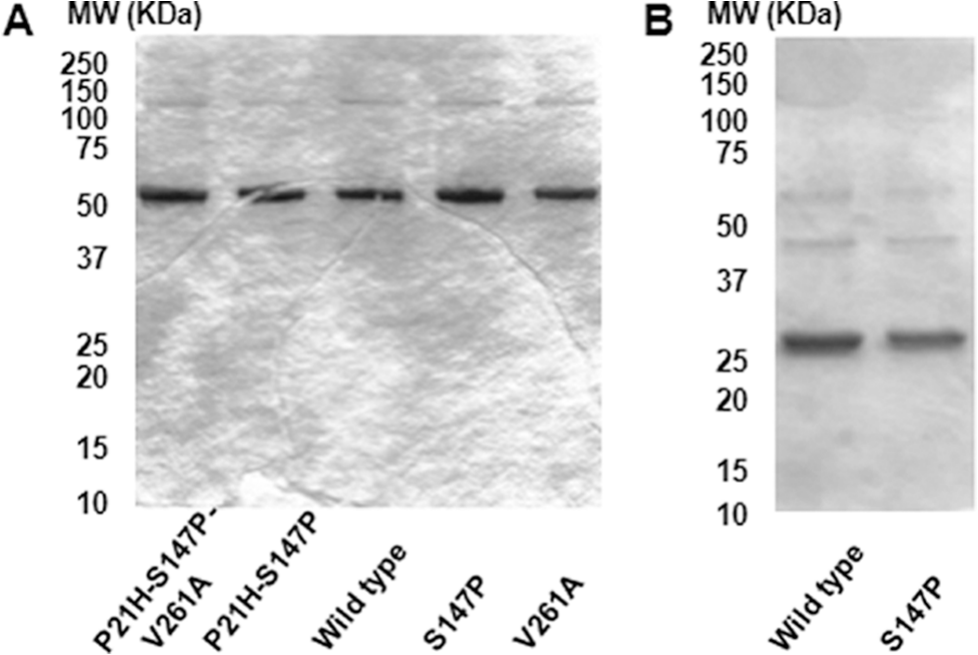
SDS-PAGE characterization of ^15^N-labeled N-terminal segments of nmMLCK1. (A) Five ^**15**^N-labeled 1-494aa proteins (ca. 3 μg each). (B) Two ^**15**^N-labeled 1-264aa proteins (ca. 3 μg each).

Extending earlier success in nmMLCK protein expression [8, 36, 37], we established a novel bacterial system for structure-oriented protein expression utilizing bacterial IMPACT system (NEB) using a C-terminal intein-chitin-binding domain (CBD) tag, which has an advantage in that the tag can be rendered traceless, i.e., readily cleavable in the presence of free thiols in the late stage of purification [28, 29]. The nmMLCK-encoding *MYLK* gene was codon-optimized by commercial gene synthesis service (Genscript, NJ) for enhanced bacterial protein expression under low temperature to promote correct protein folding and avoid aggregation. This strategy was successfully used to express and purify multiple wild type and SNP variants of the N-terminal segments of nmMLCK, unlabeled or ^15^N-labeled in two lengths, which include the 1-494aa segments and 1-264aa segments as verified by SDS-PAGE (Fig 2A and 2B) and MALDI-time of flight (TOF) MS.

### Potential application in post-translational modifications and protein-protein interactions

The IMPACT (NEB) protein expression platform exhibits an additional advantage as an appropriate fluorescent or affinity tag can be added to the C-terminus by the expressed protein ligation strategy [28, 29, 38] to facilitate various functional studies. As a proof of principle, a site-specifically biotinylated N-terminal segment 1-494aa of nmMLCK, wild type-1-494-biotin, and, upon c-Abl treatment, wild type-1-494-biotin-pTyr, were generated. As a result of high-efficiency biotinylation, the biotinylated (and Tyr-phosphorylated) protein achieved an optimal loading onto the commonly used streptavidin-coated sensor chip in a chip loading experiment of surface plasmon resonance (SPR) (S1 Fig), a prime methodology for measuring protein-protein interaction. As multiple phosphorylation sites are located within the N-terminus, a biotinylated nmMLCK protein fragment allowed for facile isolation of its kinase-phosphorylated form from a kinase reaction mixture, an advantageous strategy in SPR or other systems for examination of phosphorylated protein-protein interaction analysis. In separate experiments, we confirmed *in vitro* phosphorylation of the two 1-494aa segment variants by the catalytic domain of the c-Abl Tyr kinase (S2 Fig).

### NMR analysis of the nmMLCK 1-494aa segments reveals minimal local conformational difference between wild type and SNP variants

NMR and X-ray crystallography are major biophysical techniques that provide atomic level structural information of proteins. Unlike X-ray crystallography studies, NMR studies do not require crystallization of nmMLCK protein fragments but is limited by the size of protein that can be studied [39]. We performed 1D proton NMR experiments with an unlabeled 1-494aa segment, the wild type-1-494aa, and found such segment suitable for NMR analysis in terms of folding and stability. We subsequently carried out isotope labeling and HSQC experiments with five variants of the 1-494aa segments, including the wild type (P21-S147-V261), the H21-P147-A261 haplotype, the P147 and A261 single SNP mutants, and the H21-P147 double SNP mutant (S3 Fig). The HSQC spectra of these proteins exhibited excellent dispersion for a significant portion of ^1^H-^15^N chemical shifts, a strong indicator of the presence of a significant portion of structured regions that give dispersed and hence allow for NMR-based comparisonal analysis. A highly reproducible general pattern of HSQC spectra across tested wild type and SNP variants was also observed.

**Fig 3:**
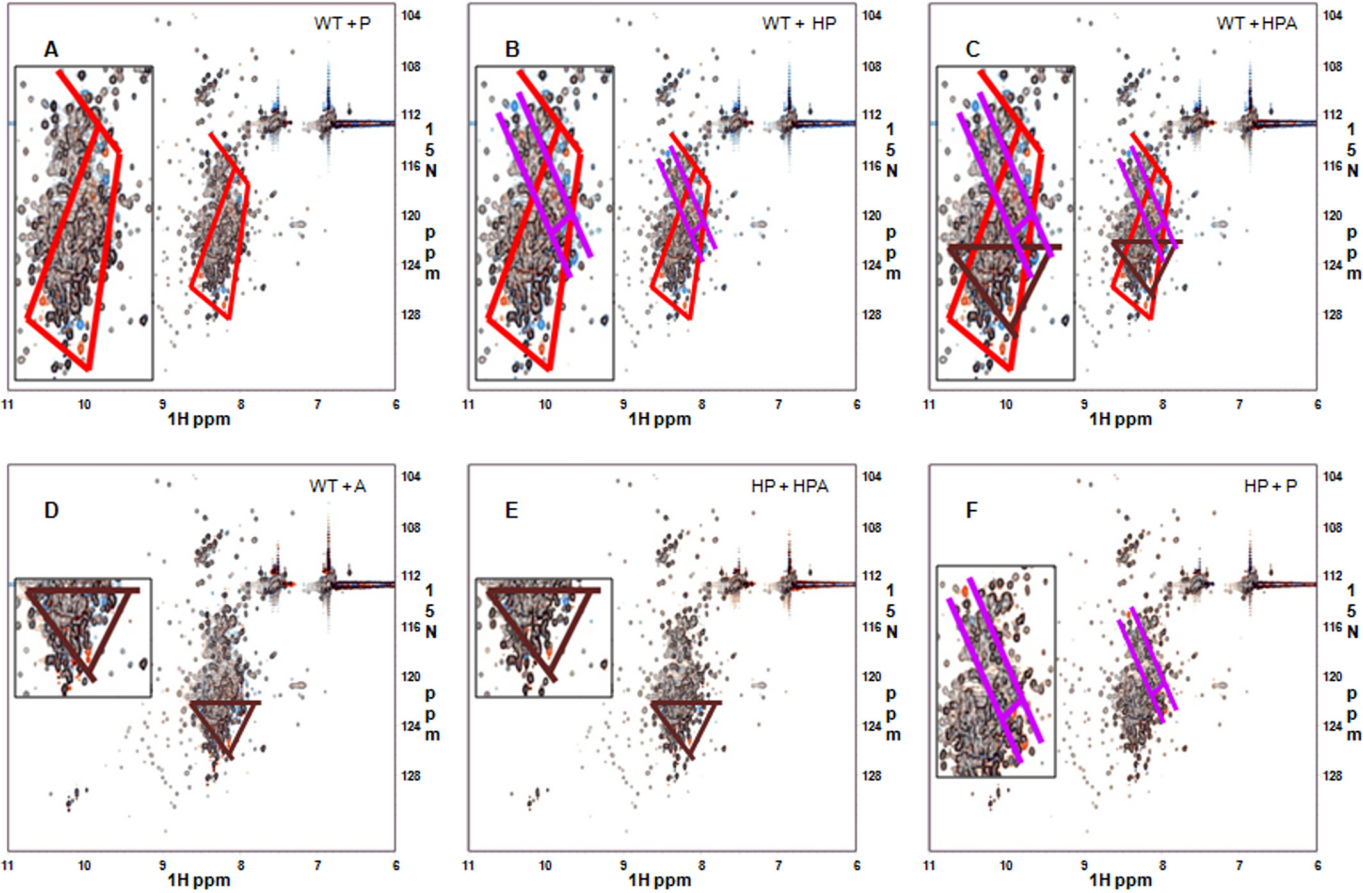
Comparison of the HSQC spectra of ^15^N-labeled 1-494aa segments of nmMLCK1. (A) Wild type and P147; (B) wild type and H21-P147; (C) wild type and H21-P147-A261; (D) wild type and A261; (E) H21-P147 and H21-P147-A261; (F) H21-P147 and P147. By superimposing the spectra of the SNP variants (the 2nd set of spectra, in *red* color) onto wild type or other SNP variants (the 1st set of spectra, in *blue* color), we have identified characteristic changes in the HSQC signals corresponding to each individual SNP mutation versus the wild type, which are indicated along red (S147P), pink (P21H) and brown (V261A) lines, respectively (see insets for more details). The characteristic patterns associated with these three SNPs suggest that these SNP mutations are indeed distant to each other in the tertiary structure and therefore cause only minor or minimal local conformational changes and independent changes in the HSQC signals.

We observed minor or minimal local conformational differences between these variants as typically expected for point mutants that do not alter overall folding or structure. By superimposing the spectra of the SNP variants (*red* color, the 2nd set of spectra) onto wild type or other SNP variants (*blue* color, the 1st set of spectra), we identified characteristic changes in HSQC signals corresponding to each individual SNP mutation versus the wild type. As expected from its mutation position in the protein and the structure of Pro, when compared to the wild type, the P147 SNP variant exhibited at least 5 appearing (*red*) and 5 disappearing (*blue*) signals, the highest number of signal changes observed among three single SNP variants (along the red lines) (Fig 3A). The H21-P147 double SNP variant, when compared to the wild type, showed 6 appearing (*red*) and 8 disappearing (*blue*) signals. Subtraction of the P147 SNP signals allowed identification of H21 SNP contributions, i.e., 1 appearing (*red*) and 3 disappearing (*blue*) signals (along the pink lines) (Fig 3B). Similarly, with the signals from both the P147 SNP and H21 SNP variants subtracted, comparison of the triple H21-P147-A261 SNP variant and the wild type resulted in identification of 2 characteristic, A261-contributed appearing signals (*red*) and 2 disappearing (*blue*) signals (along the brown lines) (Fig 3C). These characteristic (and independent from other SNPs) patterns were confirmed by subsequent analysis of additional SNP combinations and comparisons. Direct comparisons of the single A261 SNP mutant with the wild type (Fig 3D), and the triple SNP mutant with the double H21-P147 SNP mutant (Fig 3E) revealed the same characteristic pattern (along the brown lines) as Fig 3C. Lastly, superimposition of the spectra of the P147 SNP over the H21-P147 double SNP mutant (Fig 3F) resulted in a pattern almost the same as Fig 3B, but with opposite coloring. The characteristic patterns associated with these three single SNP variants suggest that these SNP mutations are distant to each other in the tertiary structure and produce only minor or minimal local conformational changes (as little as limited to that of the single amino acid, which may result in visible HSQC signal change at least for the SNP residue and a couple of spatially neighboring residues) and independent changes in the HSQC signals.

### HSQC spectra of the 1-264aa segments recapitulate the subsets of the 1-494aa segments

**Fig 4:**
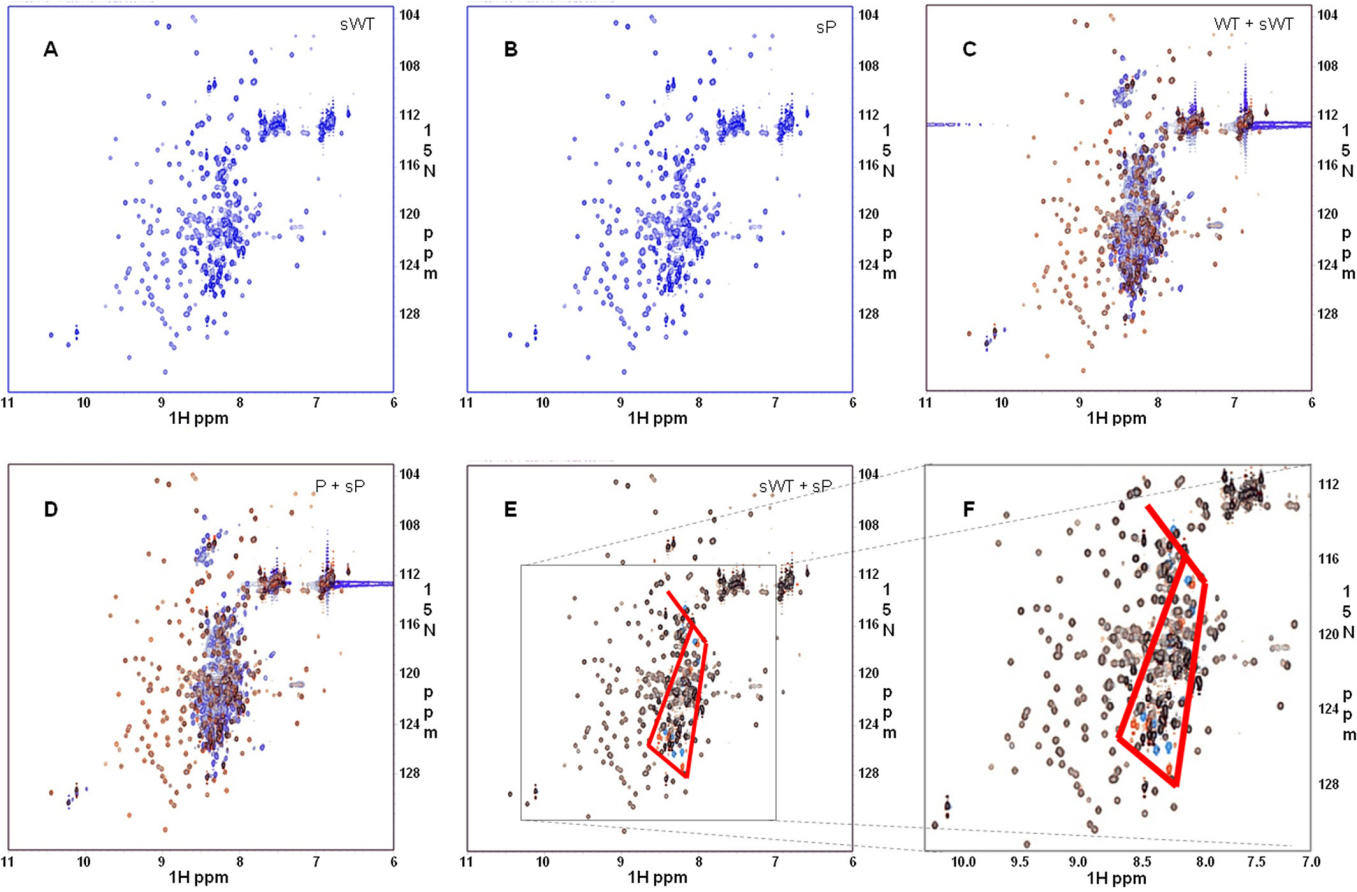
Comparison of the HSQC spectra of ^15^N-labeled 1-264aa/ 1-494aa segments of nmMLCK1. (A) and (B), the HSQC spectra of 1-264aa segments of nmMLCK1: (A) wild type; (B) P147 SNP variant. (C) to (F), the superimposition of the HSQC spectra (the 1st set of spectra shown in *blue* color and the 2nd in *red* color): (C) wild type, 1-494aa and 1-264aa; (D) P147, 1-494aa and 1-264aa; (E) 1-264aa, wild type and 147P; (F) zoom in of the squared region in (E). The HSQC spectra of the 1-264aa segments exhibited a better dispersion of ^**1**^H-^**15**^N chemical shifts, with less degenerate, better resolved signals than 1-494aa segments. Superimposition of the spectra of the 1-264aa segments onto those of their corresponding 1-494aa segments demonstrated that the spectra of 1-264aa segments are a recapitulation of the subsets of those of their corresponding 1-494aa segments, suggesting that the shorter segments each possess a structure similar to the corresponding part of their longer counterparts. The same characteristic pattern of signal changes observed for 1-494aa segments is recapitulated with better resolution by superimposition of the spectra of the 1-264aa 147P SNP mutant onto those of the 1-264aa wild type segment, suggesting that the same structural difference exist for the 1-264aa wild type and the P147 SNP mutant.

While a significant portion of the signals in the HSQC spectra of the 1-494aa segments were well dispersed, these segments were less amenable to NMR-based structural analysis due to hard-to-resolve degenerate signals in the region of 8.0–8.6 ppm in ^1^H chemical shift. Shorter segments of the 1-264aa, the wild type and the P147 SNP mutant, were next examined by HSQC, with the spectra of the two smaller proteins exhibiting excellent dispersion of ^1^H-^15^N chemical shifts, with improved signals (Fig 4A and 4B). Superimposition of the spectra of the 1-264aa segments onto those of their corresponding 1-494aa segments (Fig 4C and 4D) demonstrated that the spectra of a 1-264aa segment is a recapitulation of the subsets of those of their corresponding 1-494aa segment, suggesting that the shorter segment possesses structural similarity to the longer segment. The same characteristic pattern of signal changes as observed for 1-494aa segments (Fig 3A) is recapitulated with better resolution by superimposition of the spectra of the 1-264aa P147 SNP mutant onto those of the 1-264aa wild type segment (Fig 4E and 4F), suggesting that the same structural difference exist for the 1-264aa wild type and the 147P SNP mutant, as that of the corresponding 1-494aa segments. On the other hand, an otherwise almost identical majority of HSQC signals of the two SNP variants precludes the possibility of a global structural change across the 1-264aa segments and again indicate that any such structural change would be very minor or minimal.

To summarize our NMR results, we found overall similarity in the structures of the wild type and the SNP variants, however, characteristic, minor or minimal, local differences are present. The three SNPs each correlate with a distinguishable set of HSQC signals, suggestive of relatively independent and minimal local structural changes, with the S147P SNP variant displaying the most significant signal variation. These results were confirmed in the 1-264aa segments, with improved resolved signals for NMR-based structural determination and interaction analysis.

### Molecular modeling of the N-terminal segments of nmMLCK1

**Fig 5:**
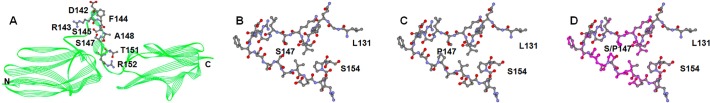
Local conformational changes in the 31-253aa segment of nmMLCK1 [template: titin Z1Z2 (PDB ID: 1ya5A)] upon S147P SNP mutation and energy minimization. (A) The S147P SNP site localized in a potentially flexible loop connecting the 1st IGc2 domain and the 2nd IGc2 domain (with side chains of some neighboring residues shown in ball and stick model). (B) to (D), a closer look at the loop connecting the 1st IGc2 domain and the 2nd IGc2 domain after energy minimization (131-154aa shown in ball and stick model): (B) the loop in the wild type; (C) the loop in the P147 SNP variant; (D) overlay of the two loops (structural changes caused by the P147 SNP mutation shown in pink).

By examining NMR and cell signaling results, we observed differential cell signaling between wild type and SNP mutants of nmMLCK1 (unpublished data) but only minor structural differences in their N-termini (by NMR). Since there are no available structures for these variants, we utilized bioinformatic and homology modeling tools to study them. In homology modeling using Swiss-Model [31], we first identified titin Z1Z2 domains (PDB ID: 1ya5A) [40] as an optimal template (sequence identity, 28.57%; QMEAN Z-score, –3.39) [32] for modeling the segment 31-253aa of nmMLCK1 that spans the first two IGc2 domains and their linker region (Fig 5A). Leveraging the HSQC results obtained, we hypothesized the P147 SNP variant to produce greater structural perturbations than other two SNP variants. Indeed, similar to NMR observation, the S147P SNP mutation induced only minor local conformational changes within the seemingly flexible loop that embraces the mutation (Fig 5B–5D). The root-mean-square deviation (RMSD) is 0.04 angstrom for the backbones of the linkage loops (137-149aa), and more reduced (0.01 angstrom) for the 31-253aa segments [33].

### Potential influence of P21H and S147P SNP mutations on the binding to 14-3-3 proteins

**Fig 6:**
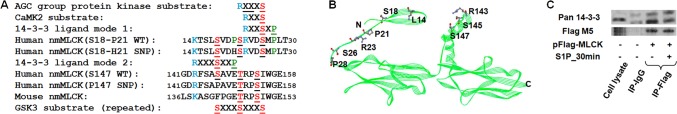
Potential involvement of S147P and P21H SNP sites in nmMLCK1 in phosphorylation-dependent binding of 14-3-3 proteins. (A) Sequence alignment of human nmMLCK1 (wild type and SNPs) with the consensus of 14-3-3 binding modes and selected protein kinase substrates as well as its murine ortholog. The “S” or “T” in *red* indicates a phophorylation site, with underline indicating a predicted site (*black* underlined) or a confirmed site (*red* underlined). The “R” or “K” in *blue* and the “P” in *green* indicate their potential involvement in key binding recognition. The “P” indicates a “P” that may be missing and hence nonessential. The “X” in *black* indicates any amino acid. The “X” indicates an “X” that may be missing and hence nonessential. The consensus of GSK3 substrate (SXXXS, the 2nd S representing a pre-existing pSer or pThr) is shown in repeat in order to align with multiple potential phosphorylation sites in nmMLCK1. While not shown, additional multiple alignments of S147, S18, and other nearby Ser residues including S16, S26, S145 and S154, are possible with the highly variable consensus substrate RXXXS of the AGC group of protein kinases that include PKA, PKG and PKC families, suggesting a complex regulation of nmMLCK via these SNP-embracing loops by different kinase-mediated phosphorylation and subsequent binding to 14-3-3 proteins. (B) Molecular modeling of the 1-252aa segment of nmMLCK1 [template: deleted in colorectal cancer (DCC) (PDBID: 3lafA)] revealing localization of S147P and P21H SNP sites in separate loops at the two ends of the single, 1st IGc2 domain (with side chains of some loop residues of interest shown), despite that the modeling of loop conformations may be of poor quality. (C) Immunoprecipitation (IP) of Flag-tagged nmMLCK1 wild type using Flag-M5 anitibody followed by western blot using pan-14-3-3 antibody indicating the binding of 14-3-3 proteins to nmMLCK1 before and after S1P stimulation. Note: In the IP result shown, the bottom 14-3-3 bands correspond to 24 kDa and the top 14-3-3 bands correspond to 27 kDa.

The observation by NMR and molecular modeling that these SNPs per se cause only minor local structural changes suggest that the key change caused by SNP variants may not be a simple 3D structural change, but may instead lie in a shift of posttranslational modification and/or protein-protein interaction. Modeling of the 31-253aa segment against the titin Z1Z2 domains suggested that S147P SNP site of nmMLCK1 is in a flexible loop that connects the 1st IGc2 domain and the 2nd IGc2 domain (Fig 5). Analysis of nmMLCK primary structure suggested that S147 in wild type (descendent allele), but not P147 in the SNP variant (ancestral allele), fits into a consensus sequence RXXXSXXP for binding to 14-3-3 proteins (mode 2), in an S147 phosphorylation-dependent fashion [41] (Fig 6A). In addition, the sequence immediately C-terminal to the 147 residue contains two Ser/Thr residues, T151 and S154, which align well with two confirmed tandem phosphorylation sites, T146 and S149, in the mouse ortholog [42, 43] (Fig 6A). T151 phosphorylation in nmMLCK would render S147 to be phosphorylated by glycogen synthase kinase-3 (GSK3), which acts efficiently on the phosphorylation-primed substrate that contains a pre-existing pSer or pThr at the 4th residue to the C-terminus [44]. Therefore S147 in the wild type (descendent allele) may differ from P147 in the SNP variant (ancestral allele) by its regulation via phosphorylation-dependent binding to 14-3-3 proteins.

Since 14-3-3 proteins may function as homo- or heterodimers [45], we were inspired to search for additional potential sites that could participate in bidentate binding to 14-3-3 proteins. Deleted in colorectal cancer (DCC), a netrin-1 receptor (PDB ID: 3lafA) [46], was selected as a different template (lower sequence identity, 25.51%; QMEAN Z-score, -3.23) in molecular modeling of the longer, 1-252aa segment of nmMLCK1. Despite that the predicted loop conformations may not be of high quality, the S147P and P21H SNP sites were localized in separate loops at the two ends of the 1st IGc2 domain (Fig 6B). Of interest, we identified two putative sites within the loop N-terminal to the 1st IGc2 domain for binding to 14-3-3 proteins in a phosphorylation-dependent fashion (Fig 6A and 6B). The P21H SNP site fits into the same consensus sequence RXXXSXXP (14-3-3 binding, mode 2), together with the nearby N-terminal Ser phosphorylation site S18 (Fig 6A), a phosphorylation site of PKA confirmed in earlier studies by our lab [18]. On the C-terminal side of the S18-P21 site, the S26-P28 site fits into a consensus sequence RXXSXP for binding to 14-3-3 proteins (mode 1) in a phosphorylation-dependent fashion [41] (Fig 6A and 6B). The S26 site also fits into the consensus sequence RXXS as a substrate of calcium/calmodulin-dependent protein kinase-2 (CaMK2) [47], which is activated similarly to MLCK by calcium/calmodulin. Notably, the phosphorylation of the S26 site by CaMK2 can prime for subsequent tandem phosphorylation of both S22 and S18 (with the tandem four-residue intervals from S26), by GSK3 (as similarly predicted above for S147 phosphorylation from a pre-existing, phosphorylated T151) (Fig 6A). In addition, the preference of proline by GSK3 [44] and the phosphorylation by the AGC group of protein kinases [48], may be involved in a complex regulation of nmMLCK via these SNP-embracing loops. Taken together, S147P and P21H SNP mutations may deviate from the wild type nmMLCK in optimal, phosphorylation-dependent, monodentate or bidentate binding to 14-3-3 proteins, hence leading to an altered nmMLCK signaling.

Binding of nmMLCK to 14-3-3 proteins was confirmed in nmMLCK immunoprecipitates (IP) from ECs overexpressing Flag-tagged nmMLCK1 (Fig 6C) using pan-14-3-3 antibody to detect 14-3-3 binding. 14-3-3 binding of nmMLCK was detected both before and after EC stimulation by S1P. In separate experiments, the addition of a 14-3-3 antagonist, BV02 [or 2-(1,5-dimethyl-3-oxo-2-phenyl-2,3-dihydro-1H-pyrazol-4-ylcarba-moyl)terephthalic acid, EMD Millipore, MA], reduced S1P-stimulated Y^464^ phosphorylation in ECs overexpressing EGFP-nmMLCK1 wild type, S147P SNP or P21H-S147P-V261A 3SNP for several minutes. This blockade of S1P-stimulated Y^464^ phosphorylation is consistent with the role of 14-3-3 as scaffolding proteins in regulating enzymatic activity and subcellular localization. However, multiple SNP- and phosphorylation-dependent 14-3-3 binding motifs within the N-terminal region suggest a potentially complex regulation of nmMLCK via 14-3-3 binding, with much more details awaiting clarification.

## Discussion

The nmMLCK isoform exerts a gate-keeper function in regulating both lung fluid balance and vascular access of inflammatory cells to lung tissues. Thus, our interrogation of nmMLCK structure, function, and regulation are of functional importance to understanding the contributions of genetic variants to inflammatory injury susceptibility. Using NMR and homology modeling, we have now defined the potential influence of two *MYLK* coding SNPs, particularly P147S, on the structure and function of nmMLCK, as impacting phosphorylation and protein-protein interaction. Further examination of homology models indicate that these two sequences are the only potential phosphorylation-dependent 14-3-3 binding sequences in nmMLCK with a serine or threonine residue well positioned in an extended loop region and thus allow high accessibility, strongly suggesting that they are responsible for the observed binding of nmMLCK to 14-3-3 proteins (Fig 6C).

**Fig 7:**
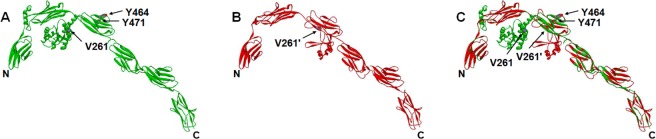
Molecular modeling of the N-terminal segments of nmMLCK1 (30-813aa) and nmMLCK2 (30-744aa) [template: titin I65-I70 (3b43A)]. (A) nmMLCK1 (*green*); (B) nmMLCK2 (*red*); (C) superimposition of nmMLCK1 (*green*) and nmMLCK2 (*red*). The positions of V261 (V261’ in nmMLCK2), Y464 and Y471 (in nmMLCK1 only) are indicated as appropriate. V261 in nmMLCK1 localizes immediately preceding the 1st alpha-helix of an alpha-beta fold which contains multiple Pro-rich motifs that may be implicated in binding to partner proteins containing SH3 domains. V261’ in nmMLCK2 localizes within the 1st beta-strand following the 2nd IGc2 domain. Y464 and Y471 are exposed at the back surface of the structures shown for nmMLCK1. A well-folded, the 2nd IGc2 domain-interacting, alpha-beta structure (ca. 253-405aa) that embraces the V261A SNP site and multiple Pro-rich loops, together with the 3rd IGc2 domain (ca. 405-505aa), in nmMLCK1, is replaced by a relatively loosely-folded structure composed of less helixes, more beta strands and more accessible Pro-rich loops (ca. 253-440aa) in nmMLCK2.

The V261A SNP is located adjacent to the 2nd IGc2 domain and at the beginning of next potentially folded structure that precedes the 3rd IGc2 domain in nmMLCK1. Although V261A has minimal influence on nmMLCK structure, our homology modeling, using I-band fragment I65-I70 from titin (PDB ID: 3b43A) [49] as the template, revealed V261 to reside immediately preceding the 1st alpha-helix of an alpha-beta fold containing multiple Pro-rich motifs potentially involved in SH3 domain binding (Fig 7A), which agrees with its localization within the disordered region of another model built by protein threading (fold recognition) [50] (Fig A in S4 Fig), despite low homology (about 20%) in this homology model. In contrast, the alanine substitution in V261A may be more favorable for folding into an alpha-helical structure and may be involved in protein folding [51–53], and eventually lead to altered nmMLCK signaling.

Additional evidence supporting the potential role of V261A SNP mutation comes from homology modeling of the shorter nmMLCK2 splice variant of nmMLCK1. The nmMLCK2 variant is the primary splice variant in ECs and lacks a 69aa-stretch containing two key Tyr phosphorylation sites, pY464 and pY471. In contrast to nmMLCK1, V261A in nmMLCK2 was found within the 1st beta-strand subsequent to the 2nd IGc2 domain (Fig 7B), which is also confirmed by its localization within the threaded model (Fig B in S4 Fig). Comparison of the N-terminal segments of nmMLCK1 and nmMLCK2 (spanning the first few IGc2 domains) reveals significant structural differences in proximity to the V261A SNP site (Fig 7C). The sequence between the 2nd and the 3rd IGc2 domains in nmMLCK1 forms a fold consisting of alpha-beta structure and Pro-rich loops that appear to interact with the 2nd IGc2 domain (Fig 7A). The same sequence in nmMLCK2 joins with the remaining sequence of the truncated 3rd IGc2 domain (missing 69aa) to form a new, less alpha-folded and more beta-folded structure, with multiple Pro-rich loops appearing more accessible for binding (Fig 7B). Superimposition of the two structures (Fig 7C), reveals a well-folded 2nd IGc2 domain-interacting, alpha-beta structure (ca. 253-405aa that includes the V261A SNP site and multiple Pro-rich loops), together with the 3rd IGc2 domain (ca. 405-505aa), in nmMLCK1, is replaced by a relatively loosely-folded structure composed of less alpha helices, more beta strands and more accessible Pro-rich loops (ca. 253-440aa) in nmMLCK2. This modeling observation is consistent with the stronger nmMLCK2-cortactin interaction observed in our previous studies. Cortactin, an actin-binding and SH3 domain-containing protein, serves as a key cytoskeletal binding partner of nmMLCK and essential barrier regulatory effector [54]. Cortactin is pp60src and c-Abl substrate, which responds to S1P and other barrier-promoting agonists by translocating to lamellipodia and membrane ruffles [2, 15, 30]. The interaction of the cortactin SH3 domain with nmMLCK is required for S1P-induced peripheral MLC phosphorylation [15]. In an *in vitro* assay, cortactin reduced nmMLCK1 binding to F-actin, whereas unphosphorylated nmMLCK1 abolishes cortactin-enhanced Arp2/3-dependent actin polymerization [54]. Therefore the cortactin-nmMLCK interaction is a critical regulator of cytoskeletal rearrangement that is necessary for S1P-mediated EC barrier enhancement [15, 30]. Cortactin binds to nmMLCK1 and nmMLCK2 with different affinities *in vitro*, with an apparent K_d_ of 1 μM for nmMLCK1 and 0.25 μM for nmMLCK2 [15], consistent with stronger binding to SH3 domain by more accessible Pro-rich motifs in nmMLCK2 suggested by modeling (Fig 7). The SH2 domain-binding motif absent in nmMLCK2 may be compensated by an enhanced binding to Pro-rich motif-binding domains such as SH3 and would subject the two splice variants to differential regulations involving not only Tyr phosphorylation but SH3 interaction as well, speculation that is consistent with our previous studies [30]. The V261A mutation, which would render the molecule more flexible, may also similarly enhance binding to partner proteins that contain SH3 (or other Pro-rich motif-interacting) domain (Fig 7). It would be interesting to examine whether the V261A mutation and the exon 11 deletion in nmMLCK2 are synergistic in this aspect.

The 14-3-3 proteins are highly conserved and ubiquitously expressed, existing as at least seven isoforms in mammals [55, 56]. These proteins play key regulatory roles in signal transduction and apoptosis by modulating the localization, phosphorylation state, stability, and molecular functions of target proteins [55–58]. For example, the association of the myosin light chain phosphatase (MLCP) with myosin II and its localization at stress fiber is down-regulated by RhoA/Rho-kinase–dependent MLCP phosphor-rylation and the resulting MLCP-14-3-3 binding [59]. In c-Abl-induced apoptosis following oxidative stress, the nuclear targeting of c-Abl is regulated by the phosphorylation of 14-3-3 zeta on Ser^184^ by JNK, in response to DNA damage [60, 61]. In addition, 14-3-3 binding and cytoplasmic sequestration of c-Abl is dependent on the phosphorylation of c-Abl on T^735^ [60, 61].

Since our previous studies indicated that c-Abl is critical to nmMLCK regulation, we speculated that 14-3-3 scaffolding proteins (with multiple isoforms) participate in nmMLCK regulation either directly or via partner proteins including c-Abl. The regulation of nmMLCK subcellular localization is not limited to N-terminal sequences as the nmMLCK association with cortactin involves the SH3 domain of cortactin as well as the cortactin- and actin-binding domains of nmMLCK [15, 54]. Of note, the EGFP-nmMLCK2Nterm fusion protein lacking actin- and cortactin-binding nmMLCK domains is virtually absent in lamellipodia, suggesting that these domains are likely required for nmMLCK translocation to lamellipodia [30]. Future studies will integrate 14-3-3 protein-mediated interactions into current knowledge of regulatory networks required for nmMLCK signaling.

To conclude our NMR and homology modeling studies, we strongly believe that inflammatory lung injury-associated SNPs in nmMLCK, namely, P21H, S147P and V261A, alter nmMLCK signaling by influencing the binding of nmMLCK to 14-3-3 proteins and SH3 domain-containing proteins protein partners (or other Pro-rich motifs). Binding of nmMLCK to 14-3-3 proteins was predicted to be variably affected by Ser/Thr phosphorylation but adversely affected by ALI- and asthma-associated SNPs. This was confirmed by immunoprecipitation and western blot studies. Our ongoing studies, employing protein expression, NMR and X-ray crystallography-based structural biology, cell signaling, and molecular modeling, are anticipated to further elucidate nmMLCK binding partnerships, and the influence of SNPs on nmMLCK-mediated cell signaling. These structure-function studies, suggesting novel mechanisms for nmMLCK regulation, may serve to confirm *MYLK* as a candidate gene in inflammatory lung disease and advance knowledge of the genetic underpinning of lung-related health disparities.

## Supporting Information

S1 Fig Surface plasmon resonance (SPR). Streptavidin (SA) chip loading of biotinylated 1-494aa-pTyr protein.
(TIF)

S2 Fig Phosphorylation of 1-494aa segments, both wild type and 147P SNP variant, by the catalytic domain of cAbl confirmed by western blot. (TIF)

S3 Fig The HSQC spectra of five ^15^N-labeled 1-494aa segments of nmMLCK1. (A) Wild type (P21-S147-V261); (B) P147 SNP mutant; (C) H21-P147 double SNP mutant; (D) H21-P147-A261 haplotype mutant; (E) A261 SNP mutant. The HSQC spectra of these proteins exhibited excellent dispersion of ^1^H-^15^N chemical shifts, a strong indicator of the presence of a tertiary structure with high likelihood for proper folding, hence suitability for NMR-based structural determination or interaction analysis. A highly reproducible general pattern of HSQC spectra across tested wild type and SNP variants was also observed.
(TIF)

S4 Fig Molecular models built for nmMLCK1 and nmMLCK2 by protein threading or fold recognition. (A) Full-length nmMLCK1; (B) Full-length nmMLCK2. Positions of key SNP residues are indicated to facilitate comparison with other models. The domain organization of nmMLCK isoforms in these models is very similar to those in homology models, except that their disordered regions are drawn in a more extended way, instead of modeling on a template.
(TIF)
