# Supplementary figures and images for: Structure–Function Analysis of the Non-Muscle Myosin Light Chain Kinase (nmMLCK) Isoform by NMR Spectroscopy and Molecular Modeling: Influence of MYLK Variants
Source: PLoS One. 2015 Jun 25;10(6):e0130515. doi: 10.1371/journal.pone.0130515 (PMC4482139; doi:10.1371/journal.pone.0130515)

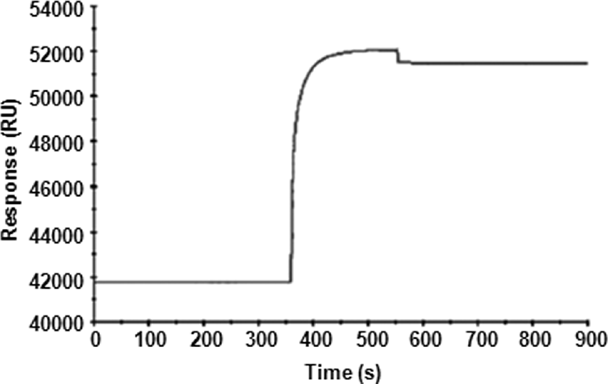

Supplement: S1 Fig — Streptavidin (SA) chip loading of biotinylated 1-494aa-pTyr protein. (TIF) [file pone.0130515.s001.tif]

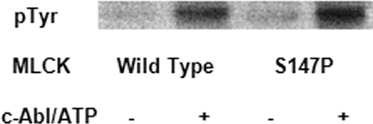

Supplement: S2 Fig — (TIF) [file pone.0130515.s002.tif]

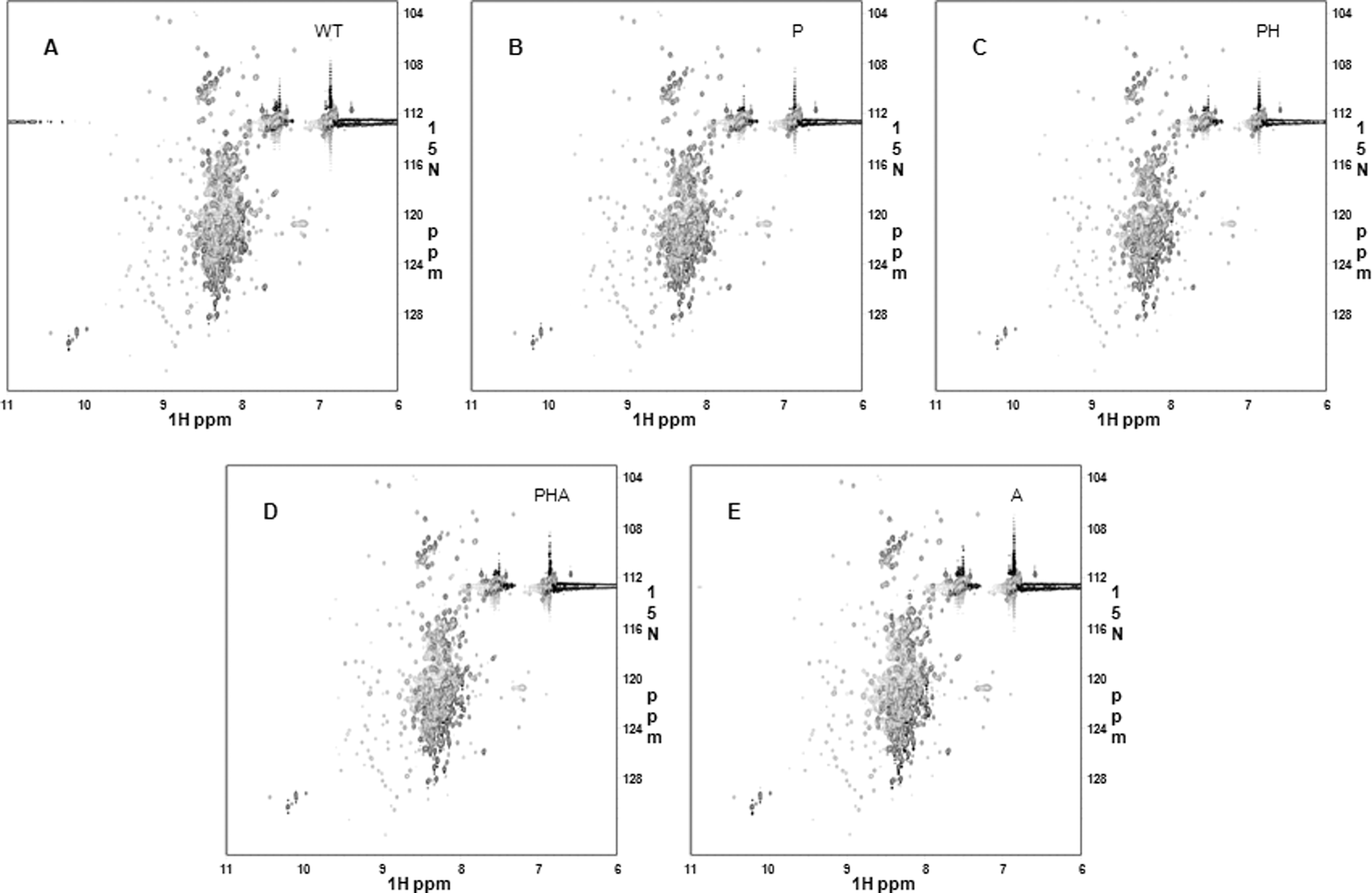

Supplement: S3 Fig — (A) Wild type (P21-S147-V261); (B) P147 SNP mutant; (C) H21-P147 double SNP mutant; (D) H21-P147-A261 haplotype mutant; (E) A261 SNP mutant. The HSQC spectra of these proteins exhibited excellent dispersion of 1H-15N chemical shifts, a strong indicator of the presence of a tertiary structure with high likelihood for proper folding, hence suitability for NMR-based structural determination or interaction analysis. A highly reproducible general pattern of HSQC spectra across tested wild type and SNP variants was also observed. (TIF) [file pone.0130515.s003.tif]

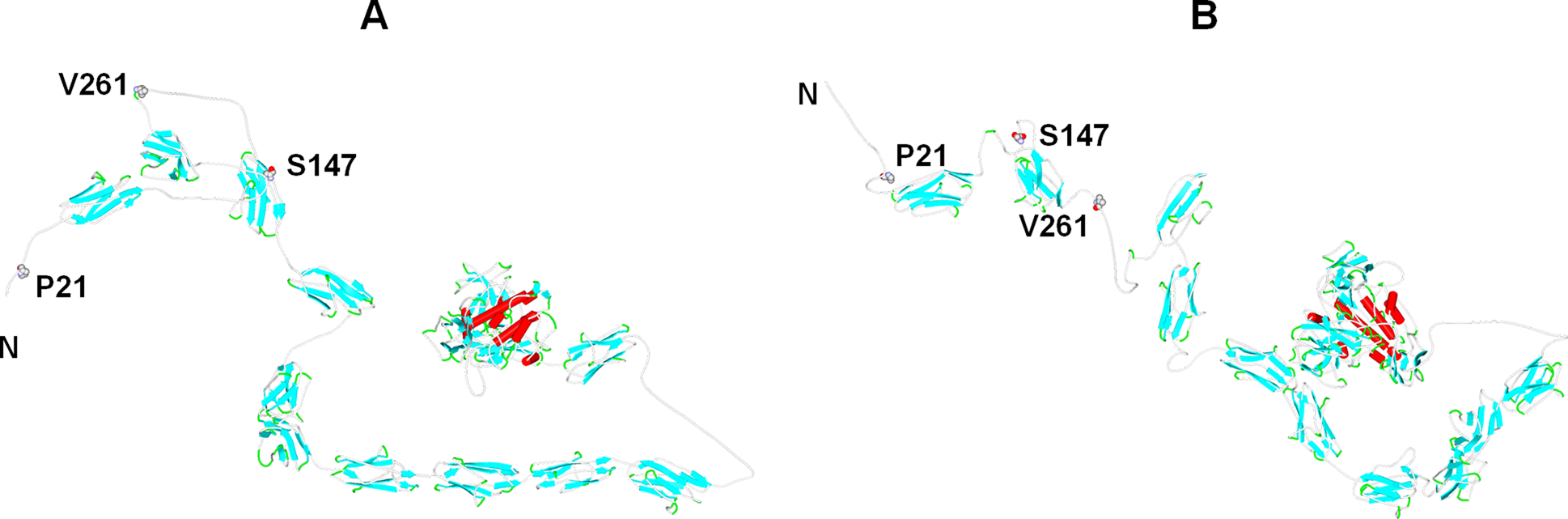

Supplement: S4 Fig — (A) Full-length nmMLCK1; (B) Full-length nmMLCK2. Positions of key SNP residues are indicated to facilitate comparison with other models. The domain organization of nmMLCK isoforms in these models is very similar to those in homology models, except that their disordered regions are drawn in a more extended way, instead of modeling on a template. (TIF) [file pone.0130515.s004.tif]
